# Omics Potential in Herbicide-Resistant Weed Management

**DOI:** 10.3390/plants8120607

**Published:** 2019-12-14

**Authors:** Eric L. Patterson, Christopher Saski, Anita Küpper, Roland Beffa, Todd A. Gaines

**Affiliations:** 1 Department of Plant, Soil and Microbial Sciences, Michigan State University, 1066 Bogue St., East Lansing, MI 48824, USA; patte543@msu.edu; 2 Plant and Environmental Sciences Department, Clemson University, 306B Biosystems Research Complex, Clemson, SC 29634, USA; saski@clemson.edu; 3 Bayer AG, CropScience Division, Weed Control Research, Building H872, 65926 Frankfurt, Germany; anita.kuepper@bayer.com (A.K.); roland.beffa@bayer.com (R.B.); 4 Department of Bioagricultural Sciences and Pest Management, Colorado State University, 1177 Campus Delivery, Fort Collins, CO 80523, USA

**Keywords:** weed genomics, herbicide resistance database, herbicide resistance diagnostics, precision herbicide resistance management, functional genomics, weed biology, weed evolution, integrated pest management

## Abstract

The rapid development of omics technologies has drastically altered the way biologists conduct research. Basic plant biology and genomics have incorporated these technologies, while some challenges remain for use in applied biology. Weed science, on the whole, is still learning how to integrate omics technologies into the discipline; however, omics techniques are more frequently being implemented in new and creative ways to address basic questions in weed biology as well as the more practical questions of improving weed management. This has been especially true in the subdiscipline of herbicide resistance where important questions are the evolution and genetic basis of herbicide resistance. This review examines the advantages, challenges, potential solutions, and outlook for omics technologies in the discipline of weed science, with examples of how omics technologies will impact herbicide resistance studies and ultimately improve management of herbicide-resistant populations.

## 1. Introduction

Reference genome assemblies have enabled many advances in our understanding of gene function and the linkages between the genome and phenome. Modern plant biology has become quantitative, systems-oriented, and predictable. The fields of genomics, transcriptomics, proteomics, and metabolomics—collectively referred to as ‘omics’—describe the component parts of the biological system that lead to the presentation of traits. Profound developments have been realized in model plant and crop species where the genome and associated omics systems have led to new biological understanding and application [1]; however, the question remains—how can omics and associated systems-scale biology contribute to our understanding of herbicide resistance and ultimately help improve weed management? Fundamentally, this is a question of how omics discoveries can translate into applied outcomes and innovations. Within weed science, genomics and transcriptomics have been the most utilized of the various omics techniques and are the focus of this review. Proteomics and metabolomics are also emerging as potential areas of research for herbicide resistance [2,3,4]; however, the full potential of omics techniques has not yet been realized [5].

**Figure 1:**
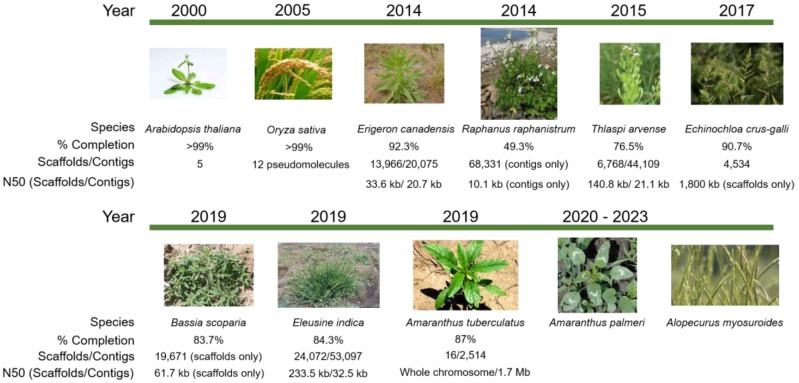
Timeline of weed genome assembly in comparison to the model plants *Arabidopsis thaliana* and rice. The first weed genome assembled to chromosome-level scaffolds is *Amaranthus tuberculatus* [7], for which scaffolding was completed by aligning with a related crop genome, *Amaranthus hypochondriacus* [8]. Other weeds with assembled genomes in various stages of completeness include *Erigeron canadensis* [9], *Thlaspi arvense* [10], *Echinochloa crus-galli* [11], *Bassia scoparia* [12], and *Eleusine indica* [13]. Assemblies for *Amaranthus palmeri* and *Alopecurus myosuroides* are in progress. Image sources: Arabidopsis, https://www.eurekalert.org/multimedia/pub/159783.php; field pennycress, https://www.agweb.com/article/pennycress-gets-in-the-middle-chris-bennett; horseweed, https://oregonstate.edu/dept/nursery-weeds/weedspeciespage/horseweed/horseweed_habit.html; wild radish, http://science.halleyhosting.com/nature/plants/4petal/must/raphanus/raphanistrum.html; barnyardgrass, http://swbiodiversity.org/seinet/taxa/index.php?taxon=2915&taxauthid=1; kochia, photo courtesy of Phil Westra, CSU; goosegrass, https://www.invasive.org/browse/detail.cfm?imgnum=5387295; Palmer amaranth, https://www.mda.state.mn.us/plants/pestmanagement/weedcontrol/noxiouslist/palmeramaranth; waterhemp, https://agfaxweedsolutions.com/2019/02/11/waterhemp-scores-again-new-resistance-found/; blackgrass, https://www.fwi.co.uk/arable/crop-management/weed-management/blackgrass/how-to-use-integrated-methods-to-control-blackgrass; rice, http://aaasjournal.org/rice-fields-chemical-physical-properties-implications-breeding-strategies/rice-plant/.

Several weed genomes have been completed to various levels of assembly completeness (Figure 1). Plans are in progress to rapidly and substantially expand the availability of weed genomics resources [6]. While the costs for sequencing are on a continuous decline and computational capacity is increasing, major challenges remain to fully realize the potential of omics and their contribution to improved weed management. In this review, we present what omics studies have already contributed to herbicide resistance and weed management, explore the challenges for omics in weeds, identify translational aspects of model systems, discuss the trajectory and impact of integrating omics in weed science, and propose a road map for where the discipline should go in the future to harness the power of omics for improved herbicide resistance management.

## 2. Challenges Specific to Weed Science

Omics research in weed science faces several challenges, some specific to weed science and some generic to the entire field of omics research. Several of these will be addressed with new discoveries and technologies that are currently being developed, while others may need a concerted effort by the weed science community to address. We will lay out several of these challenges and some possible solutions that may arise to meet them.

### 2.1. Managing Omics Datasets

The size and complexity of omics datasets being generated necessitates excellent database resources including large data storage, data backups, easy access, and data manipulation tools both in weed science and omics research at large. Several toolkits for genome databases have been developed and successfully implemented with support from both private and public sectors. For example, Tripal was developed with support from various academic and government funding agencies and is freely available for download [14]. Tripal was designed to streamline and simplify the process of omics database generation and organization, even in an online format [14]. Tripal also allows for the integration and use of several important bioinformatics tools such as BLAST, InterPro, gene function enrichment analysis, etc., an approach employed by several plant genome groups such as the Cucurbit Genomics Database [15] and the Genome Database for Rosaceae [16]. Other database services for omics can be licensed from the private sector, e.g., CropPedia by KeyGene (https://www.croppedia.com/).

Aside from establishing a contemporary platform for data housing and manipulation, deciphering a complex, quantitative phenotype still remains a challenge. Data from the genome, epigenome, transcriptome, proteome, and metabolome can now be collected from the same plant, and even single cells in some cases. A primary goal is to understand the latent relationships among the omics datasets to derive a comprehensive understanding of the underlying biology. In the example above, taking a holistic approach (e.g., collection of different omics datasets) offers power and resolution in comprehensively understanding the cellular and molecular components (and their interactions) [17]; however, integrating discrete experimental results is still difficult because of the inherent differences in the data [18]. Furthermore, there are limitations in omics technologies that are confounded by the complex nature of living systems [19]. As data integration techniques and strategies continue to advance, holistic interpretation of systems data will improve our biological understanding of complex phenotypes.

### 2.2. Genome Annotation

Another significant challenge facing the entire genome community is efficient and accurate annotation of reference genome assemblies and eventual pan genomes. Homology-based gene annotation pipelines, such as Maker [20] and Blast2GO [21], rely heavily on well-annotated, phylogenetically close relatives to the species of interest for gene model evidence. These tools perform even better with the availability of transcriptome datasets that are representative of key tissue sources selected across the developmental life cycle. Many weed species of interest do not reside close enough to a genomically-enabled neighbor species to be useful in homology-based gene annotation. Frequently, the closest species to weeds with sequenced genomes reside in distant plant families or even orders. Typical gene annotation strategies include the use of several popular prediction algorithms, such as SNAP [22], Augustus, GenesFH, GeneMark, Glimmer, and others. These algorithms can be trained with species specific data, manual curation, and consensus predictions extracted with programs such as EVidenceModeler [23]. In any case, weed species are often described as having exceptional genomes, with dynamic genomic plasticity and unique genetic content that can rapidly adapt and endow extreme phenotypes [7,12,24,25].

Another profound gap exists between computational prediction of genes and gene function, and validation of gene expression and its role and interaction with components of the omics system. A primary goal in weed science is to develop modern tools that leverage omics datasets to enable the study and verification of gene function *in situ*. A current constraint in closing this gap is the lack of curated and well-maintained germplasm banks and the lack of gene editing and transformation protocols. The lack of such tools prevents functional studies on genes and gene families, limiting the ability to fully harness the power of genomics for weedy traits.

### 2.3. Diversity of Evolutionary Strategies in Weeds

Weed genomics has other challenges that are specific. One of the biggest challenges is the number of species being studied globally. It seems impossible to select a weed species (or even a handful of weed species) that represent the diversity in weed science (not only the phylogenetic diversity but also the diversity of weed management problems). Currently, the priority species for genomics research are those that have the largest economic impact; species such as *Amaranthus palmeri, Alopecurus myosuroides*, *Echinochloa crus-galli,* and *Lolium* spp. However, these species are not always the most tractable for basic biology research. For instance, *Amaranthus palmeri* is dioecious, confounding the development of specific populations for population-level genetic analysis; or the fact that *Alopecurus myosuroides* has an exceptionally large, repeat-rich genome (~3.5 Gb) with high amounts of heterozygosity; or polyploid genomes like *Echinochloa crus-galli*. A ‘model weed’ approach could be used to deeply investigate fundamental questions about the great diversity of weedy traits and variation in evolutionary strategies found in weeds [26,27,28], while new resources may be developed for specific applications to compare results across multiple weed species.

One proposed explanation for the way in which some weed species continue to be dynamic in the face of elastic environmental pressures (avoiding genetic bottlenecks) is through maintenance or generation of genetic diversity [29]. Genetic diversity is critical for adaptation, and is perhaps, a key component in understanding the origins of traits and speciation; however, distinguishing genetic diversity from environmentally-induced phenotypic variability and linking phenotypes to genes poses several challenges. First and foremost is the ability to find, maintain, and accurately characterize lines with quantifiable heritability for traits of interest. Without consistent, well-characterized phenotypes, finding the genes through traditional methods (test crosses, genome-wide association studies (GWAS), QTL-seq, etc.) becomes a much more difficult task. Secondly, highly homogenous lines are desired as the starting point for genome assembly projects. Highly heterogeneous genomes are much more difficult to assemble and typically result in lower contiguity and completeness with a higher degree of inaccuracy [12]. To compensate, extra sequencing and haplotype phasing is typical in the assembly process, requiring additional time and expense. Furthermore, genetic studies that take advantage of segregating populations comprised of recombinant inbred lines (RILs) [30] offer high degrees of resolution and discrete QTL windows. For weed species that are obligate outcrossers (e.g., dioecious *Amaranthus* spp., self-incompatible *Lolium* spp.), the development of homozygous populations is not possible, leaving mapping resolution to be defined by half-sibling segregating populations and/or GWAS approaches where population structure confounds mapping resolution.

Weed scientists have ambitious goals to study complex traits in weeds, such as abiotic stress, seed germination, and non-target site resistance (NTSR) [6,31,32]. A major challenge is that these traits will be investigated across multiple weed species representing diverse plant families. No one model weed can represent the full range of life history traits and biology present across weeds. An interesting example of a (generally, but not always) highly quantitative complex trait is NTSR to herbicides. The study of NTSR is further complicated by the many combinations of weed species and registered herbicides for which multiple resistance mechanisms are possible [31]. The genetic basis and inheritance of herbicide resistance can be complex [33,34], such as NTSR mechanisms that are quantitative between populations and between individuals in a given population [35,36,37,38,39]. Elucidating the basis of NTSR to one herbicide in one species is not necessarily extensible to other herbicides and other species. In addition, NTSR mechanisms can endow cross-resistance to multiple herbicides with different sites of action [40,41,42], and single plants can contain multiple different NTSR mechanisms [36,43]. This further confounds the use of omics strategies to disentangle the underlying genetic mechanisms. Likewise, weed species in general display an interesting disposition of resilience for complex abiotic traits that are of agronomic importance such as drought, heat, salt, and cold resilience, as well as seed longevity and many others. Dissecting these traits on a molecular basis can prove to be difficult without modern omics approaches.

## 3. Addressing Challenges by Looking at Other Disciplines

Many research disciplines that work in model systems have already begun to fully exploit the decreased costs of next generation sequencing (NGS). Looking at the diverse ways researchers working in model systems are using omics technologies in their respective fields can provide established tools and templates to address the unmet needs of the weed science community.

### 3.1. Method Standardization for Utilizing NGS in Weed Science

With more and more researchers utilizing NGS, the need for quality and methods standards that enable comparisons between studies becomes paramount. First and foremost, weed scientists need access to reference lines used in NGS studies, especially since the high usage of herbicides worldwide has made it more difficult to obtain purely susceptible populations. For some species, reference susceptible lines are in common use such as the ‘Roth’ line of *Alopecurus myosuroides* maintained by Rothamsted Research Institute, which has never had herbicide exposure in the past 150 years [44], the *Lolium rigidum* line VLR1 from Victoria, Australia [45], and the susceptible *Bassia scoparia* line 7710 from Colorado [12,46]. Likewise, references exist for resistant weed populations like the established *A. myosuroides* “Peldon” [44], the *L. rigidum* lines VLR69 [47] and SLR31 [42], and the first *Amaranthus palmeri* population reported to be resistant to glyphosate [48,49]. Distribution of these reference lines is currently only on an ad hoc basis by contacting the labs that maintain them. The greater challenge is to capture the diversity of resistance mechanisms and combinations and maintain their availability over a long period for future studies. Reference lines should be stored with institutions like the USDA National Laboratory for Genetic Resource Preservation (USDA-NLGRP), which already houses a broad germplasm collection, or an arrangement similar to the NSF-funded Sequence-Indexed Library of Insertion Mutations for *A. thaliana* [50] that propagates seed for distribution to the community. Easily accessible reference lines can then be used for sequencing projects, dose response experiments for herbicide sensitivity or fitness penalty studies, population genetics studies, as control groups, or to test gene function. In the future, we hope to see homozygous recombinant inbred lines (RIL) or multi-parent mapping populations in weed science for the identification of more complex quantitative trait loci (QTL). For situations in which homozygous lines may be difficult to produce (e.g., self-incompatible species, dioecious species, multiple resistance mechanisms), we encourage the production and availability of multiple reference populations.

Due to the high demand from researchers working on model systems, NGS data analysis can be performed through several publicly available platforms, for example the NSF-funded CyVerse with data storage and bioinformatic tools through the Discovery Environment web interface [51], or the Galaxy project [52]. Furthermore, NSF-funded labs have produced easy to use online tools like the Genome Sequence Annotation Server (GenSAS) that provides a pipeline for *de novo* gene prediction and whole genome structural and functional annotation [53]. More tools that are weed science specific may need to be developed or adapted from other existing tools; for instance, a database of consistent annotations and gene ontologies. The Antibiotic Resistance Ontology (ARO) service [54] and the Cytochrome P450 homepage [55] have shown how important proper annotations are to provide consistent vocabulary for genes, which form much of the foundation of genomic bioinformatics.

Currently, omics techniques used for weeds are limited in scope, usually to a pair of samples and just a few individuals per population. In the future, more NGS studies will be available for meta-analyses that can provide insights into more complex evolutionary questions and the basic mechanisms driving complex traits like metabolic herbicide resistance. Additionally, we may soon be able to perform whole genome sequencing from many individuals of a single species for genome-wide association studies (GWAS) and pangenome analysis, which will provide key information about genetic variability and evolutionary history of individuals and populations. Similar to human genotypic ancestry services, the more individual genome information is available for weeds, the better genetic relatedness, movement patterns, and invasion biology can be understood.

### 3.2. Improving Herbicide Resistance Diagnostics with Omics

We predict that improved resistance diagnostics in combination with field history data will allow for field-tailored precision weed control recommendations that avoid unnecessary one-size-fits-all treatments and improve risk prediction tools. Improved diagnostics and precision mapping might also support or refute zero tolerance approaches in the case of new and agriculturally troublesome herbicide resistance mutations.

Currently, the International Survey of Herbicide Resistant Weeds [56] is the main database for herbicide resistant weeds and provides an extensive collection of new resistance reports and genomic DNA sequences that encode for herbicide targets in various weed species. The antibiotic resistance field maintains the Resistance Map, which provides interactive data on antibiotic use and resistance patterns worldwide and predicts resistance trends [57]. The Comprehensive Antibiotic Resistance Database (CARD) collects antibiotic resistance genes and associated proteins and takes the idea a step further to also provide information on antibiotics, resistance mechanisms, antibiotic targets, associated phenotypes, and tools to analyze molecular sequences. It also predicts putative antibiotic resistance genes from unannotated but assembled contigs and their prevalence from sequenced genomes [54,58]. These data are also essential to weed scientists to ask questions such as when and where are the first cases of resistance, how widespread are they, by what mechanism of resistance it is conferred, what is the agricultural relevance for the grower, and how are herbicides being used on a global scale? We foresee the need for weed resources such as weedscience.org expanding in scope to include more reporting of resistance mechanism (e.g., target-site resistance, TSR, and NTSR) and being partially modeled based on resources developed by microbiologists.

### 3.3. Improved Gene Function Validation for Herbicide Resistance Mechanisms

The increase in sequencing efforts to investigate mechanisms of resistance has led to an increase in the identification of candidate driver genes that correlate with resistant phenotypes. Unfortunately, many studies do not continue to functionally validate these candidate genes and the actual cause for resistance remains undetermined. In successful validation studies, researchers have utilized *Agrobacterium tumefaciens*-mediated transformation of candidate genes in model plant systems, such as *Arabidopsis thaliana* [59,60,61,62], tobacco (*Nicotiana benthamiana*) [63], rice calli (*Oryza sativa*) [64,65], transgenic rice [66,67], or budding yeast (*Saccharomyces cerevisiae*) [68]. However, most weed science studies fall short of functionally validating identified genes due to lack of investment to date in stable plant transformation methods for weeds. Plant transformation is an area where method development is urgently needed.

Plant gene function can be investigated using transient expression systems to either knock out or overexpress a candidate gene variant. Currently, there are several alternative techniques available in non-model species for the investigation of gene function by RNA interference (RNAi) such as virus-induced gene silencing (VIGS) [69,70]. Relevant to herbicide resistance, this technique has been recently utilized to silence CYP749A16 in trifloxysulfuron-tolerant cotton [71] and to silence a GST gene cluster in *Verticillium* wilt-resistant cotton [63]. Plants can also be inoculated with modified virus alone, resulting in transcription of anti-sense RNA and subsequent target mRNA cleavage, such as the barley stripe mosaic virus system used in cereals [72]. Alternative techniques to suppress target mRNA by direct topical applications of anti-sense silencing oligos have been developed such as small interfering RNAs (siRNAs) in a complex with a protein carrier [73], high-pressure spraying of double-stranded RNA (dsRNA) [74], or through simple application of long dsRNA [75]. In contrast to reverse genetics approaches that knock out gene function by anti-sense transcript silencing, gain of function due to candidate gene variants can be assessed with transient expression in plants using promoter-targeted RNA-directed DNA methylation (in cases where DNA methylation can affect gene transcription) [76] and transient infection with *Agrobacterium* to express a candidate gene [77].

Most gene function studies in model systems have used alternative transfer DNA (t-DNA) or transposon insertional mutagenesis to create mutant plants (gene knock-outs) for phenotype screening where plants with interesting phenotypes were further characterized for the affected gene(s). These techniques require the production and maintenance of a large amount of germplasm as well as huge resource input. This is only feasible when a large community is working on a single species (e.g., *Arabidopsis*). For weed scientists it may be more viable to take a targeted approach for gene knockouts using gene editing techniques like zinc finger nucleases (ZFNs) [78,79] or transcription activator-like effector nucleases (TALEN) [80]. Additionally, gene editing using clustered regularly interspaced short palindromic repeats/CRISPR-associated protein 9 (CRISPR/Cas9) guided by small RNA instead of proteins for sequence-specific DNA cleavage [81] may be the quickest way to achieve targeted gene editing. CRISPR systems have been shown to work both transiently or stably and with high efficiency and specificity [82]. The weed science community would benefit greatly from implementing these techniques to validate candidate gene function; however, as for other approaches to study gene function, investment in plant transformation methods for weed species is needed to fully enable gene editing in weeds.

## 4. Using Current and Future Omics Tools to Improve Herbicide Resistant Weed Management

Potential applications of genomics for improving applied weed control have been reviewed [5,6,27,28,83]. A striking example of technology that could advance weed management is the gene drive system [84]. Gene drives that could result in species extinction may be unfeasible for regulatory and/or public acceptance reasons. However, some weedy traits may be excellent gene drive targets to reduce the impacts of weeds. For example, if genomics can identify the basis of extreme allergenicity in weeds (e.g., ragweed species), a gene drive system could target elimination of the allergen from populations. If genomics can identify the basis of seed dormancy, a gene drive system could lead to greater synchronization of germination. Tumbleweeds require the development of an abscission layer at the base of the plant to break off, tumble, and disperse seed. A gene drive system could potentially eliminate the abscission layer trait from a population, reducing spread of the tumbleweed seeds.

Externally-applied gene silencing techniques to manipulate gene expression and potentially reverse herbicide resistance mechanisms are another application of new knowledge gained from genomics [85]. However, major challenges remain to utilize externally-applied gene silencing in plants, specifically difficulties in stability, delivery, and efficacy of gene silencing oligonucleotides [74,75]. The resources from expanded weed genomics efforts will be crucial to design effective gene silencing triggers with maximum specificity to target species and with minimal off-target effects, both in the target organism and in non-target organisms.

Improved understanding of pathogen response pathways in weeds could lead to opportunities for improved biocontrol. For example, pathogens could be engineered to be more virulent on weeds but not on crops [86,87]. Gene drive systems could be combined with bio-control to spread susceptibility to a pathogen within a weed population, potentially enabling long-term suppression of populations without further intervention. Weeds that are alternate hosts for crop pathogens could be targeted with gene drive or gene silencing to eliminate their ability to serve as alternate hosts.

The UK BioBank provides an example from the biomedical science field as to how large-scale availability of genotypic and phenotypic data on thousands of individuals can democratize genomics and make possible the discovery of the genetic basis of many diseases and traits in humans [88]. For the model plant *Arabidopsis*, full genome sequences and phenotyping data exist for more than 1000 lines, along with databases of corresponding RNA-Seq gene expression data and gene knockout mutation phenotypic effects [89]. We envision a similar weed biobank database empowering research on weeds across the world, consisting of reference genomes for multiple species, phenotypic data contributed from collaborators around the world, and genome wide genotype data sets that are publicly available and can be mined to discover the basis of quantitative traits, complex herbicide resistance mechanisms, and other traits of interest in weeds. A weed biobank for GWAS will be complemented by other tools from quantitative genetics, such as utilizing F2 mapping for herbicide resistance traits and abiotic stress tolerance traits. The integration of quantitative genetics with phenotyping including metabolomics, proteomics, and transcriptomics on segregating individuals will initially enable markers associated with traits of interest, and ultimately identify genomic regions and specific genes controlling the traits. In addition, as in cancer therapy [90], genomic diagnostics might help to choose the best herbicide combination(s) to mitigate the evolution of NTSR, in particular metabolic resistance.

## 5. Where Is Weed Omics Going?

Looking ahead to the next five to 10 years, we see several applications for weed omics. Large scale, high-throughput detection of known resistance mutations is possible using targeted amplicon NGS, bringing down the cost of genotyping and increasing the scope of available information [91]. The precision to identify resistant genotypes at low frequency within field-scale management units will enable improved management recommendations specific for growers and their unique situation of resistance mechanism(s), frequency, crop rotation, soil type, etc. The detection of low frequency resistance will enable early warning systems, both for individual growers and within regions. The use of metadata from digital agriculture will enable integration of field history and geospatial data on weed populations to further inform best practice recommendations for growers.

Like the standards proposed in Section 2 for defining and characterizing the phenotype of herbicide resistance, we envision the same standards to define and report herbicide resistance based on characterized mutations in candidate genes. Currently, resistance is defined according to biological criteria, primarily greenhouse dose responses, which has pros (reliable, not dependent on specific mechanism), but it also has cons, including the cost and time required. Additionally, the current resistance definitions in use consider resistance to be defined only when resistant individuals are at a high frequency in a population. The common term used is biotype, which is not necessarily an accurate term for many of the reports in the resistance database when there may be mixtures of different resistance mechanisms within a population (e.g., TSR and NTSR). We ask to consider whether a few highly resistant individuals within a population of mostly sensitive individuals should have a definition (e.g., early stage resistance), as this initially rare resistance frequency is when active measures can be taken to slow the increase in resistance. Improved diagnostics (faster, cheaper, more individuals tested) will enable early detection of resistant individuals within populations, and corresponding management measures to be prescribed. In-field diagnostics may have utility to provide rapid information for grower decision making, similar to how various rapid PCR techniques can be used to identify plant pathogens in the field [92].

We argue that resistance databases should accept molecular criteria to report known, well-characterized cases of resistance, both for TSR and NTSR (when the genetic basis is known). Improved resistance testing with rapid markers and database tracking is possible with modern molecular biology. Resistance cases are likely underreported in databases because, for example, reporting the next observation of acetolactate synthase (ALS) resistance has little incentive for researchers to conduct laborious and expensive assays, while ALS resistance can be easily diagnosed with molecular markers for target-site mutations. The ease in identification and reporting should help address the current bias in data for prevalence of common herbicide resistance mechanisms, data that will be important for the herbicide discovery industry.

In addition to utilization of molecular markers, resistance databases should be further improved through advances from omics technologies. More technologies should be developed to diagnose known resistance mechanisms, including nucleic acid probes, antibodies, and metabolite screens. More knowledge gaps exist for NTSR, with a few examples of known genetic variants for metabolic resistance characterized to date in weeds [61,64,93], but with many more cases of metabolic resistance to be discovered. With an improved understanding of metabolic resistance genes and pathways, transcriptional and/or protein markers can be screened as a diagnostic panel, in which the presence of defined subsets of markers indicates a sample is positive for metabolic resistance, similar to what is currently performed in cancer diagnostics [94,95]. Such a diagnostic panel has already been shown for weeds, with different sets of transcriptional markers for cytochrome P450s and other NTSR genes able to differentiate metabolic resistant and susceptible *Lolium* field populations collected in France [96,97].

We propose a system to classify metabolic resistance genes, such as cytochrome P450s and GSTs, by their capacity to metabolize the known herbicide structures. To achieve this will require both discovery and validation of genes in these gene families utilizing genomics, as well as cloning these genes into heterologous systems (e.g., yeast, *Arabidopsis*) to determine their metabolic activity on each herbicide of interest. Undertaking this objective will require considerable investment and coordination, due to the high number of cytochrome P450s genes in plants and their sequence and functional divergence across plant families [98]. Collectively, this information will inform management by shedding light on cross-resistance patterns due to metabolism, as well as enable testing of compounds in development and those yet to be discovered for their susceptibility to metabolism by resistant weeds.

## 6. Summary

The weed omics era is enabling translational research to bridge from basic science to field applications, by linking systems-scale science to applied science for practitioners. The rise of digital farming and dense geospatial data will enable prediction tools for the occurrence and spread of herbicide resistance within fields and across landscapes. This metainformation will improve diagnostics as well as provide greater insight into the factors driving selection for various resistance mechanisms. Machine learning will lead to algorithms to select the best options from chemical and non-chemical control technologies [99]. Weed omics will contribute to better define these prediction tools and associated algorithms. These benefits of weed omics will be more challenging to realize for farms not utilizing the advanced data science approaches necessary for implementation of digital farming.

While there are substantial challenges today to apply omics to weed science, the coming years will see development of new approaches to help overcome these challenges. As the increase in data acquisition continues to coincide with the development of new statistical approaches to systems biology, what seems like insurmountable obstacles now may soon be trivial issues. Whole genome sequencing projects have evolved from large-scale international efforts to routine tasks often undertaken by an individual lab. For example, obtaining a high quality reference genome of a heterozygous plant would not have been possible only a decade ago, and now the International Weed Genomes Consortium has pledged to generate 10 in only a few years [6], in addition to several key species recently completed outside this collaboration [7,12].
